# Endophytic bacterial community structure and diversity of the medicinal plant *Mirabilis himalaica* from different locations

**DOI:** 10.1007/s42770-023-01149-1

**Published:** 2023-11-03

**Authors:** Erhao Zhang, Yazhou Lu, Rundong Zhao, Xiu Yin, Jie Zhang, Benxia Yu, Min Yao, Zhihua Liao, Xiaozhong Lan

**Affiliations:** 1 The Provincial and Ministerial Co-Founded Collaborative Innovation Center for R & D in Tibet Characteristic Agricultural and Animal Husbandry Resources, The Center for Xizang Chinese (Tibetan) Medicine Resource, Joint Laboratory for Tibetan Materia Medica Resources Scientific Protection and Utilization Research of Tibetan Medical Research Center of Tibet, Tibet Agriculture and Animal Husbandry University, Nyingchi, 860000 Tibet China; 2 College of Life Science and Agronomy, Zhoukou Normal University, Zhoukou, 466001 Henan China; 3 Chongqing Academy of Chinese Materia Medica, Chongqing, 400065 China; 4 Jiangxi Institute for Drug Control, NMPA Key Laboratory of Quality Evaluation of Traditional Chinese Patent Medicine, Nanchang, 330029 Jiangxi China; 5 Key Laboratory of Eco-Environments in the Three Gorges Reservoir Region, Ministry of Education, Chongqing Engineering and Technology Research Center for Sweetpotato, School of Life Sciences, Southwest University, Chongqing, 400715 China

**Keywords:** *Mirabilis himalaica*, Tibetan traditional plant, Plant endophytes, Illumina sequencing, Geographic conditions, Vegetal tissues

## Abstract

Endophytic bacteria play important roles in medicinal plant growth, abiotic stress, and metabolism. *Mirabilis himalaica* (Edgew.) Heimerl is known for its medicinal value as Tibetan traditional plant; however, little is known about the endophytic bacteria associated with this plant in different geographic conditions and vegetal tissues. To compare the endophytic bacterial community associated with this plant in different geographic conditions and vegetal tissues, we collected the leaves, stems, and roots of *M. himalaica* from five locations, Nongmu college (NM), Gongbujiangda (GB), Zhanang County (ZL), Lang County (LX), and Sangri County (SR), and sequenced the 16S rRNA V5-V7 region with the Illumina sequencing method. A total of 522,450 high-quality sequences and 4970 operational taxonomic units (OTUs) were obtained. The different tissues from different locations harbored unique bacterial assemblages. Proteobacteria and Actinobacteria were the dominant phyla in all the samples, while the dominant genera changed based on the different tissues. The endophytic bacterial structures in the leaf and stem tissues were different compared to root tissues. Redundancy analysis (RDA) showed that the endophytic bacterial community was significantly correlated with pH, available phosphorus (AP), total phosphorus (TP), total nitrogen (TN), and soil organic matter (SOM). These findings suggested that the geographic conditions, climate type, ecosystem type, and tissues determined the endophytic bacterial composition and relative abundances. This conclusion could facilitate an understanding of the relationship and ecological function of the endophytic bacteria associated with *M. himalaica* and provide valuable information for artificial planting of *M. himalaica* and identifying and applying functional endophytic bacteria.

**Supplementary Information:**

The online version contains supplementary material available at 10.1007/s42770-023-01149-1.

## Introduction

*Mirabilis himalaica* (Edgew.) Heimerl, also known as *Oxybaphus himalaicus* Edgew, is a perennial herb distributed mainly on the Qinghai-Tibet Plateau. The root of *M. himalaica* is one of the most common medicines in Tibet and has been used in dozens of folk drugs for treating nephritis edematous, lumbago, renal calculus, arthralgia, and uterine cancer [1]. Chemical components of the roots of *M. himalaica* include rotenoids, phenylpropionates, flavonoids, phenylpropionate derivatives, steroids, organic acids, and anthraquinones [2–5]. Modern pharmacological studies have shown that the components of *M. himalaica* have inhibitory activities on tumor growth in xenografts of HepG2 cells and exhibit cytotoxic effects against A549 and HeLa cells [3, 6]. While previous studies of *M. himalaica* have focused mainly on its chemical components and medicinal value, little is known regarding the structure and function of the endophytic bacterial community of *M. himalaica*.

Endophytic bacteria are a group of microorganisms that inhabit healthy plant tissues without causing pathogenic reactions [7]. In recent decades, studies have shown that plant endophytes play a crucial role in host growth, development, stress resistance, synthesis, and pathogen biocontrol [8, 9]. In addition, previous studies have shown that plant endophytes, such as endophytic actinobacteria and *Streptomyces*, have antimicrobial, anticancer, and antidiabetic activity potential [10, 11]. Endogenous bacterial communities can catalyze the transformation of medicinal components, and endophytes have been used to transform notoginseng and ginsenoside [12, 13]. Therefore, isolating and identifying endophytic bacteria may provide a reference for applying functional endophytic bacteria from herbal materials.

Endophytes are affected by the host, environment, geographic conditions, and plant tissues. Previous studies have shown that the diversity of endophytic bacteria varies substantially in different cultivars of some plants [14–16]. For example, only 34.2% of operational taxonomic units (OTUs) were shared in two wild rose species with different powdery mildew susceptibilities [17]. In cassava cultivars with different tolerances to root rot, the community compositions were shown to be diverse, and the enrichment of functional microorganisms was different; in Kyoto Encyclopedia of Genes and Genomes (KEGG) analysis, the “Environmental adaptation” and “Infectious diseases: Parasitic” categories were significantly enriched in the tolerant and susceptible cultivars, respectively [18]. Geographic conditions have been shown to significantly affect the endophytic bacterial diversity of *Pennisetum sinese* from different locations [19], and the endophytic bacterial communities of tree peony and *Pyrus ussuriensis* have been shown to vary in different tissues [20, 21]. Therefore, analyses of the diversity and community structure of endophytes from different locations and tissues are effective for exploring critical and potentially functional microorganisms.

Endophytic bacteria can be characterized by using high-throughput sequencing technology, which provides unprecedented insight into the features of these microorganisms [22, 23]. Thus, high-throughput sequencing technology can be used to reflect the diversity and community structure that likely exist in nature [23]. Here, we comparatively analyzed the bacterial endophytic communities from five locations through Illumina sequencing technology to provide valuable information for identifying and applying functional endophytic bacteria from herbal materials.

## Materials and methods

### Sample collection

The root, stem, and leaf samples of *M. himalaica* from five locations selected at random to represent different ecosystem types of *M. himalaica* (natural ecosystem, semi-natural ecosystem, and artificial ecosystem) along the Yarlung Zangbo River were collected in our study. *M. himalaica* of Gongbujiangda County (GB) and Sangri County (SR) grew in sandy soil that belonged to natural ecosystems undisturbed by human factors, *M. himalaica* of Lang County (LX) grew in sandy soil that belonged to semi-natural ecosystem disturbed by road construction, monoculture of *M. himalaica* of Nongmu college (NM) grew in humus soil, while, the monoculture of *M. himalaica* of Zhanang County (ZL) grew in sandy soil. All samples were collected in Yarlung Zangbo River Basin, Tibet, China, in August 2019 (Table 1). To ensure that the experiment was representative, at each sampling point, the same growth phase (2 year old) and healthy, undamaged tissues from 15 plants were sampled according to the five-point sampling method [24]. All samples were cut down with sterile scissors and well mixed to form one composite sample. The composite sample from each tissue was divided into three replicates. All samples were immediately placed in liquid nitrogen until DNA extraction.

**Table 1:** Geographical sources and sample codes

Sampling site	Nongmu college (NM)	Gongbujiangda County (GB)	Zhanang County (ZL)	Lang County (LX)	Sangri County (SR)
Latitude	29° 40′ 06″ N	29° 53′ 36″ N	29° 18′ 39″ N	28° 57′ 20″ N	29° 13′ 38″ N
Longitude	92° 20′ 20″ E	93° 18′ 02″ E	91° 18′ 06″ E	93° 22′ 42″ E	92° 22′ 04″ E
Altitude (m)	2941	3389	3580	3210	3558
Annual average temperature (◦C)	8.5	8.3	8.25	11	6
Annual average precipitation (mm)	654	808.3	419.7	475	370
Ecosystem type	Artificial ecosystem	Natural ecosystem	Artificial ecosystem	Semi-natural ecosystem	Natural ecosystem

### Physicochemical analysis of rhizosphere soil

Rhizosphere soil samples from different locations were collected and filtered with a 2-mm sieve, and the pH, soil organic matter (SOM), total nitrogen (TN), available nitrogen (AN), total phosphorus (TP), available phosphorus (AP), total potassium (TK), and electrical conductivity (EC) were measured accordingly. The pH values were tested with a pH meter in a soil–H_2_O suspension (1:5, w/v). The SOM concentration was measured by the potassium dichromate method [25]. The TN and AN concentration were extracted by the Kjeldahl method [26]. The TP concentration was determined using the Mo-Sb colorimetric method, and the TK concentration was determined by the atomic absorption spectrophotometry method [27]. The AP concentration was determined by the reporting method [28]. The EC of the soil was determined by the electrode method [29].

### DNA extraction and sequencing

The samples were first washed in running tap water to remove the surface clumps and then surface-sterilized with 75% ethanol for 1 min and 2% sodium hypochlorite for 3 min. The samples were then rinsed with sterile distilled water and dried on sterilized paper [30]. The efficiency of sterilization was tested by cultivating plant fragments on Luria–Bertani (LB) plates to confirm the absence of any bacterial growth. Total DNA was extracted from surface-sterilized tissues using a plant DNA kit (Qiagen, Germantown, MD, USA) according to the manufacturer’s instructions, and the quality of the extracted DNA was verified by 1% gel electrophoresis and ultraviolet spectrophotometry (NanoDrop ND-2000). The fragment of the bacterial 16S rRNA V5-V7 region was amplified using the primers 799F (5′-AACMGGATTAGATACCCKG-3′) and 1193R (5′-ACGTCATCCCCACCTTCC-3′) for bacterial community analysis. PCRs were performed in a total volume of 25 μL containing 2.5 μL 10 × PCR buffer, 1 μL of forward and reverse primer (10 μM), 1 μL 5 mmol/L dNTPs, 0.25 μL rTaq (5.0 U/μL), 1 μL DNA template, and ddH_2_O to a final volume of 25 μL. The PCR program was conducted as follows: 95 °C for 5 min; 35 cycles of 95 °C for 30 s, 55 °C for 30 s, and 72 °C for 45 s; and a final extension at 72 °C for 10 min. The PCR products were verified by 1% gel electrophoresis and purified with the MinElute PCR purification kit (Qiagen) according to the manufacturer’s protocol and quantified by using QuantiFluor™-ST (Promega, USA). Paired-end sequencing (2 × 300 bp) was carried out on the Illumina MiSeq platform at Majorbio, Shanghai, China. The Illumina sequencing data obtained from this study were deposited in the Sequence Read Archive (SRA) database of NCBI under number PRJNA689931.

### Sequence processing and analysis

The raw data generated from the Illumina MiSeq platform were overlapped and merged using FLASH [31] and then filtered by mothur [32] to remove low-quality sequences, mismatched sequences, primer sequences, and sequences that were too long (≥ 500 bp) or too short (≤ 200 bp). Moreover, UCHIME [33] was used to filter out the chimera sequences and mitochondrial and chloroplast sequences. Finally, the remaining sequences were clustered into OTUs of 97% similarity using USEARCH [23]. The OTU sequences were annotated by the Ribosomal Database Project (RDP) classifier based on the SILVA database [34].

### Statistical analysis

Alpha diversity indexes of bacteria were analyzed using mothur [32], including the Chao1 index, Shannon index, Simpson index, and Shannoneven index. The relative abundance and beta diversity were analyzed by QIIME [35]. Venn diagrams were drawn by Venn diagram of software R based on the OTU abundance to display the number of common and unique OTUs. Principal component analysis (PCA) figures were created by the “ade4” package of R (version 3.3.1) based on Bray–Curtis dissimilarities to examine community dissimilarity. Heatmaps and redundancy analysis (RDA) beta were drawn and analyzed by R (version 3.3.1). Functional prediction of the bacterial community based on 16S rRNA was performed using PICRUSt1. The differences in alpha diversity indexes, physicochemical properties, and functional profiles were analyzed by ANOVA (one-way analysis of variance), using Dunn’s multiple comparison tests and Kruskal–Wallis test. All analyses were conducted in SPSS 22.0 [36].

## Results

### Analysis of soil physicochemical properties

The rhizosphere soil physicochemical properties were examined at the five locations (NM, GB, ZL, LX, SR), as shown in Table 2. The GB, ZL, and LX rhizosphere soil were alkaline (pH values, 7.60–8.44), the NM sample was acidic (pH values, 6.62), and the SR sample was neutral (pH values, 7.22). The TP and AN concentrations of the LX and SR rhizosphere soil were significantly higher than those of NM, GB, and ZL. The TK, TN, and SOM concentrations of the LX rhizosphere soil were significantly higher than those of NM, GB, ZL, and SR. The EC in the GB sample was significantly higher than that in other samples. The AP concentration in the NM sample was significantly higher than that in other samples, but similar in GB, ZL, LX, and SR samples. In the natural ecosystem, the properties (excepting for AP and pH) presented significantly different values between GB and SR. In the artificial ecosystem, the properties (excepting for AN and SOM contents) presented significantly different values between NM and ZL. Overall, the soil physicochemical properties were different in the different locations; these results indicated that soil physicochemical properties were influenced by environment and human factors.

**Table 2:** The physicochemical properties of the rhizosphere soil of *M. himalaica* from the different locations

Physicochemical factors	Samples				
	NM	GB	ZL	LX	SR
TP (g/kg)	0.770 ± 0.009 b	0.559 ± 0.018 c	0.501 ± 0.007 d	0.976 ± 0.027 a	0.958 ± 0.039 a
TK (g/kg)	5.797 ± 0.091 c	9.773 ± 0.446 b	2.993 ± 0.182 d	13.687 ± 0.399 a	5.759 ± 0.308 c
TN (g/kg)	0.771 ± 0.112 d	2.319 ± 0.072 c	0.124 ± 0.006 e	6.417 ± 0.132 a	3.442 ± 0.182 b
AN (g/kg)	0.145 ± 0.007 b	0.152 ± 0.003 b	0.150 ± 0.004 b	0.205 ± 0.005 a	0.220 ± 0.014 a
pH	6.620 ± 0.117 c	7.697 ± 0.120 b	8.437 ± 0.070 a	7.600 ± 0.116 b	7.217 ± 0.046 b
EC (μs/cm)	42.833 ± 11.578 e	129.433 ± 4.677 a	54.500 ± 0.361 d	105.800 ± 1.901 b	65.833 ± 1.627 c
AP (mg/kg)	29.187 ± 1.687 a	13.533 ± 0.277 b	16.194 ± 3.497 b	17.988 ± 1.039 b	15.829 ± 1.839 b
SOM (g/kg)	7.581 ± 0.695 d	38.713 ± 3.189 c	7.682 ± 2.262 d	137.777 ± 6.929 a	74.857 ± 1.216 b

Values are means ± standard error (*n* = 3). Statistical significance was calculated using Dunn’s test. The same letter represents no significant difference; the different letter represents significant difference (*P* < 0.05)

### Analysis of sequencing data and microbial diversity

After processing and standardizing according to the minimum sequence number (11,610), a total of 522,450 high-quality sequences with an average length of 376 bp were obtained from 45 samples (Table 3). A total of 4970 OTUs were obtained based on 97% similarity. Rarefaction curves tended to plateau with increasing sequencing depth in all tissue samples (leaf, stem, and root) from the five locations (ZL, SR, LX, NM, GB) (Supplementary Fig. S1), suggesting that the libraries reflected most of the bacterial diversity. A total of 1193 OTUs were shared among all the samples and represented 24.00% of the total OTUs (Supplementary Fig. S2A). A total of 170, 318, and 289 OTUs were shared in the root, stem, and leaf samples from different locations, respectively (Supplementary Fig. S2B, C, and D). These results showed that the OTUs in the same tissue from different locations were different and the distribution of OTUs was influences by environment and human factors.

**Table 3:** The number of OTUs and microbial community richness and diversity indexes of leaf (L), stem (S), and root (R) samples collected from five locations (ZL, SR, LX, NM, GB) at a 97% similarity threshold

Sample	Chao1	Shannon	Simpson	Shannoneven
ZLL	808.76 ± 50.56 bc	4.537 ± 0.149 b	0.029 ± 0.002 cd	0.702 ± 0.010 c
SRL	1694.10 ± 86.57 a	6.108 ± 0.092 a	0.006 ± 0.001 e	0.847 ± 0.013 a
LXL	1178.40 ± 207.04 ab	5.449 ± 0.211 a	0.025 ± 0.006 cd	0.784 ± 0.014 b
NML	314.29 ± 17.32 d	3.429 ± 0.182 c	0.096 ± 0.020 b	0.601 ± 0.026 cd
GBL	910.04 ± 33.89 b	4.779 ± 0.383 b	0.029 ± 0.001 c	0.723 ± 0.047 c
ZLS	813.13 ± 100.50 bc	4.233 ± 0.253 b	0.052 ± 0.011 bc	0.656 ± 0.027 cd
SRS	769.99 ± 96.68 bc	5.046 ± 0.542 ab	0.029 ± 0.030 cd	0.771 ± 0.075 bc
LXS	770.86 ± 17.92 bc	4.808 ± 0.193 b	0.045 ± 0.011 bc	0.731 ± 0.025 c
NMS	546.97 ± 117.24 cd	3.860 ± 0.117 bc	0.074 ± 0.001 b	0.630 ± 0.004 cd
GBS	820.72 ± 60.80 bc	4.489 ± 0.271 b	0.045 ± 0.017 bc	0.687 ± 0.041 c
ZLR	709.16 ± 71.423 c	4.589 ± 0.032 b	0.024 ± 0.001 d	0.736 ± 0.011 c
SRR	817.24 ± 69.179 bc	4.250 ± 0.213 b	0.039 ± 0.008 c	0.682 ± 0.024 c
LXR	631.88 ± 14.136 c	2.868 ± 0.773 c	0.239 ± 0.091 a	0.448 ± 0.117 d
NMR	950.01 ± 75.75 b	4.370 ± 0.127 b	0.034 ± 0.005 c	0.681 ± 0.012 c
GBR	876.67 ± 32.78 b	4.849 ± 0.043 b	0.020 ± 0.004 d	0.775 ± 0.007 c

Values are means ± standard error (*n* = 3). Statistical significance was calculated using Dunn's test. The same letter represents no significant difference; the different letter represents significant difference (*P* < 0.05)

### Endophytic community diversity

Endophytic community diversity was calculated using mothur. The *α* diversity indexes of all samples showed difference. The richness indexes (Chao1 indexes) and diversity indexes (Shannon and Simpson indexes) of the SRL sample were significantly higher than those of other samples, but similar with LXL (Table 3). In the same ecosystem types and tissues, the richness and diversity indexes were significantly different between NM and ZL leaf and root samples; those of GB and SR leaf samples were significantly different, but similar in root samples. The evenness indexes (Shannoneven indexes) of SRL were higher than those of other samples; however, there were no significant differences between ZL, NM, and GB leaf and root samples; interestingly, the richness, diversity, and evenness indexes were not significantly different in the stem samples of different locations. These results indicated that the multiple environment factors affected the change of endophytic bacteria of *M. himalaica*.

### Endophytic community composition

**Fig. 1:**
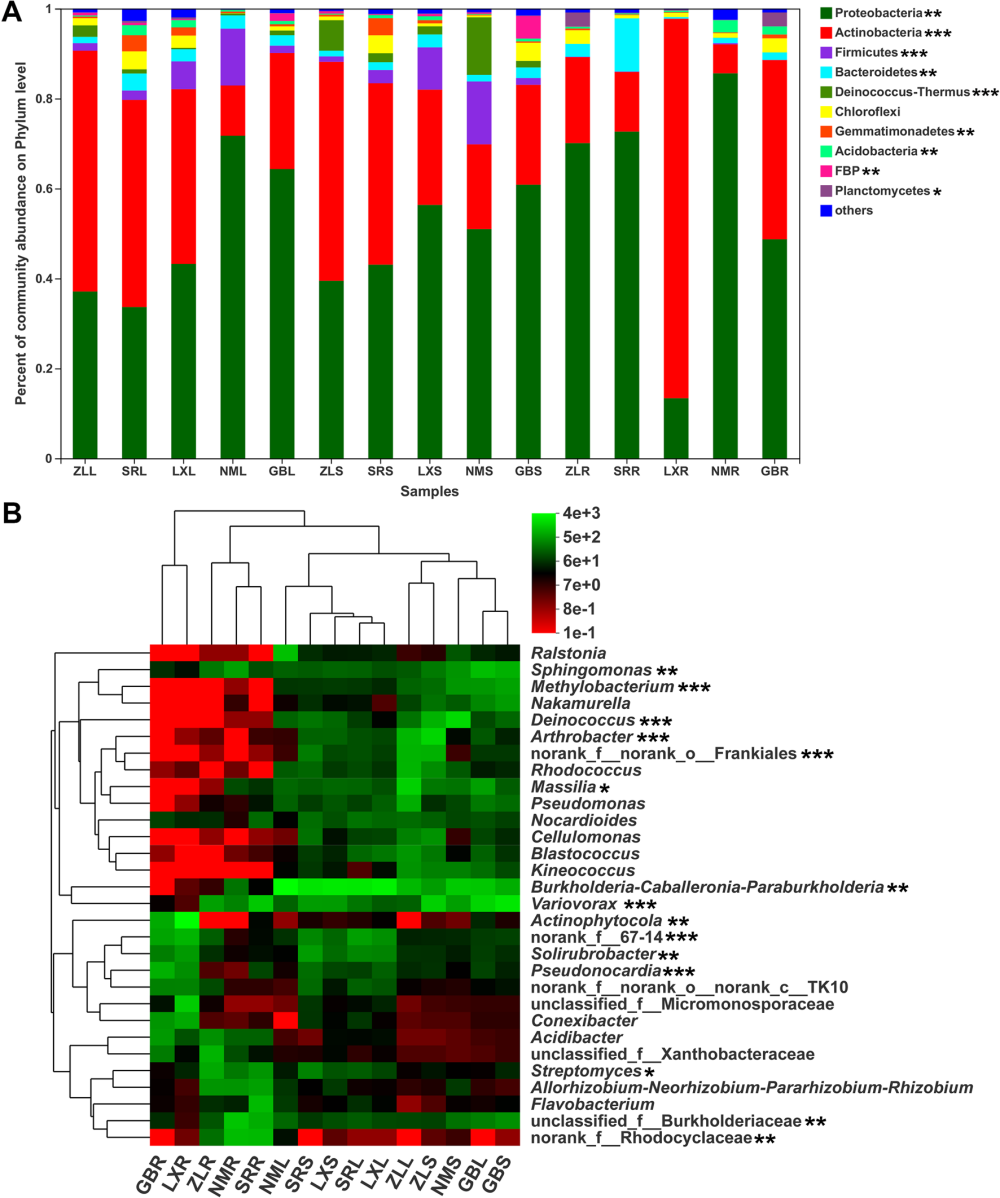
The relative abundance of the most dominant bacteria associated with leaf (L), stem (S), and root (R) tissue of *M. himalaica* collected from five locations (ZL, SR, LX, NM, GB) at the phylum (**A**) and genus levels (**B**). Statistical significance was calculated according to the Kruskal–Wallis *H* test. *P < 0.05; **P < 0.01; ***P < 0.001

Across all the samples, a total of 4970 OTUs were classified into 35 phyla, 84 classes, 242 orders, 485 families, and 1118 genera by the Ribosomal Database Project (RDP) classifier based on the SILVA database. There were a total of 10 phyla and 118 genera (relative abundance ≥ 1.0%) among all the samples, which included 81 known genera and 37 unclassified genera. Proteobacteria and Actinobacteria were the dominant phyla in all the samples, accounting for 13.19–85.62% and 6.43–81.86% of the total phyla, respectively (Fig. 1A). In the leaf tissues, Actinobacteria was more abundant in the ZL sample than in NM samples (*P* < 0.01), but similar in other samples. Gemmatimonadetes was more abundant in the SR sample than ZL, NM, and GB samples (*P* < 0.01), but similar in LX, ZL, NM, and GB samples. Acidobacteria was more abundant in the SR  sample than in the ZL sample (*P* < 0.05); however, there were no significant differences among ZL, LX, NM, and GB. In the stem tissues, Proteobacteria was more abundant in the GB than in the ZL sample (*P* < 0.05), but similar in other samples. Actinobacteria was more abundant in the ZL than in LX, NM, and GB samples (*P* < 0.05), but similar in other samples. FBP was more abundant in GB than in other samples (*P* < 0.05). In the root tissue, Proteobacteria was significantly depleted in the LX compared with other samples (*P* < 0.001). Actinobacteria was more abundant in LX than in other samples (*P* < 0.001), but similar in ZL, SR, and NM samples. Bacteroidetes was more abundant in SR than in other samples (*P* < 0.001), but similar in other samples. Acidobacteria was more abundant in NM and GB than in other samples (*P* < 0.05), but similar in ZL, SR, and LX samples. These results showed that geography affected bacterial community composition. Furthermore, Firmicutes was more abundant in leaves and stems than in roots (*P* < 0.05). Deinococcus-Thermus was more abundant in stems than in leaves and roots (*P* < 0.05). Planctomycetes was more abundant in roots than in leaves and stems (*P* < 0.05). These results showed that the host niche impacted on bacterial community composition.

At the genus level, the *Burkholderia-Caballeronia-Paraburkholderia* (10.85–35.26%) was the predominant genus in the leaf tissues, except for in the ZL sample, in which the predominant genus was *Massilia*, representing 13.26% of the total genera. In the stem tissue samples, *Burkholderia-Caballeronia-Paraburkholderia* (20.28–22.89%) was the dominant genus in the SR and LX samples, and *Variovorax* was the dominant genus in the GB sample, accounting for 21.33% of the total genera; the dominant genus in the NM samples was *Deinococcus*, representing 16.33% of the total genera; the *Arthrobacter* was the dominant genus in the ZL sample, accounting for 15.02% of the total genera. In the root tissue samples, *Streptomyces* (5.92%), *Variovorax* (11.15%), *Actinophytocola* (34.22%), *Sphingomonas* (5.03%), and *Pseudonocardia* (6.76%) were the dominant genera in the ZL, SR, LX, NM, and GB samples, respectively (Fig. 1B). At the top 30 genera, a total of 15 bacterial genera were significantly different among the different location-associated tissues, such as *Variovorax* was more abundant in GB-associated tissues than in other locations (*P* < 0.05); *Sphingomonas* was more abundant in GB-associated tissues than in LX, SR, and ZL locations (*P* < 0.05); unclassified_f_Burkholderiaceae was more abundant in NM than in LX and ZL locations (*P* < 0.05); *norank_f_67-14* was more abundant in LX than in NM and ZL locations (*P* < 0.05); *Arthrobacter* was more abundant in ZL than in other locations (*P* < 0.01); and *Solirubrobacter* was more abundant in LX than in NM and LX locations (*P* < 0.01). In the different tissues, a total of 15 bacterial genera were significantly different in the different tissues, such as *Deinococcus* was more abundant in stem tissues than in leaves and roots (*P* < 0.05), *Arthrobacter* and *Methylobacterium* were more abundant in stem tissues than in roots (*P* < 0.05), *Streptomyces* and *Pseudonocardia* were more abundant in root tissues than in leaves (*P* < 0.05), and *Rhodococcus* and *Pseudomonas* were more abundant in leaf tissues than in roots (*P* < 0.05). These results indicated that the bacterial community composition and the relative abundances were different and the geographical and host niche affected bacterial community composition.

The core endophytic microbiota for the different tissues was analyzed by Venn diagrams at the genus level. A total of 865 genera were obtained from the leaf tissue samples from the different locations; among these, the number of genera was highest in the LX leaf tissue samples (583) and lowest in the ZL leaf tissue samples (399). There were 331 core bacterial genera that coexisted in at least 4 different locations, accounting for 38.26% of the total genera. The most abundant genera were *Burkholderia-Caballeronia-Paraburkholderia*, *Massilia*, *Sphingomonas*, *Pseudomonas*, *Blastococcus*, *Bacillus*, *Bradyrhizobium*, and *Variovorax* (Supplementary Fig. S3A). Similarly, from the 929 genera in the stem tissue samples, we identified 329 genera as core bacteria, accounting for 35.41% of the total genera in the stem tissue samples. The highest number of genera was found in the LX stem tissue samples (701), while the lowest was found in the ZL samples (405); *Burkholderia-Caballeronia-Paraburkholderia*, *Variovorax*, *Deinococcus*, *Arthrobacter*, *Bacillus*, *Bradyrhizobium*, *Methylobacterium*, and *Sphingomonas* were the abundant genera (Supplementary Fig. S3B). The core endophytic bacteria for the root tissue samples were also explored, and we identified 219 genera as core microbiomes, accounting for 41.63% of the total genera (526); the top five genera were *Streptomyces*, *Acidibacter*, *Steroidobacter*, *Sphingomonas*, and *Pseudonocardia* (Supplementary Fig. S3).

**Fig. 2:**
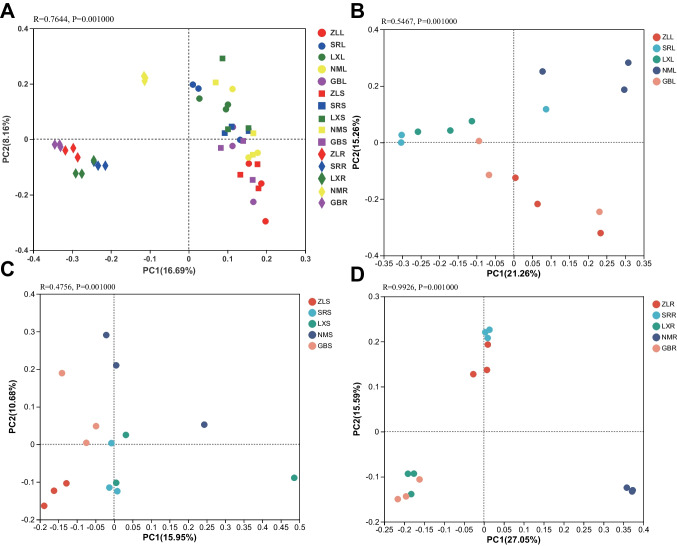
PCoA based on the relative abundance of OTUs, illustrating the differences in bacterial community composition associated with leaf (L), stem (S), and root (R) tissue of *M. himalaica* collected from five locations (ZL, SR, LX, NM, GB). **A** Different locations and tissues. **B** Leaf tissues of different locations. **C** Stem tissues of different locations. **D** Root tissues of different locations

To analyze the endophytic bacterial communities between *M. himalaica* at the five locations, principal coordinate analysis (PCoA) combined with ANOSIM was conducted to illustrate the variation in the endophytic bacterial communities in the different samples. PCoA revealed that geographical locations significantly affected the composition of bacterial community (*R* = 0.7644, *P* < 0.01) (Fig. 2A). The effects of locations on root tissues (*R* = 0.9926, *P* < 0.01) were stronger than in leaves (*R* = 0.5467, *P* < 0.01) and stems (*R* = 0.4756, *P* < 0.01) (Fig. 2B–D). The samples were clustered into two groups: group 1 consisted of leaf and stem tissues, which were represented by circle and square icons, respectively, and group 2 consisted of root tissues, represented by diamond icons. To some extent, the bacterial compositions of the GB and ZL samples were more similar, while those of the LX and SR samples were more similar (Supplementary Fig. S4).

### Relationship between environmental factors and endophytic communities

**Fig. 3:**
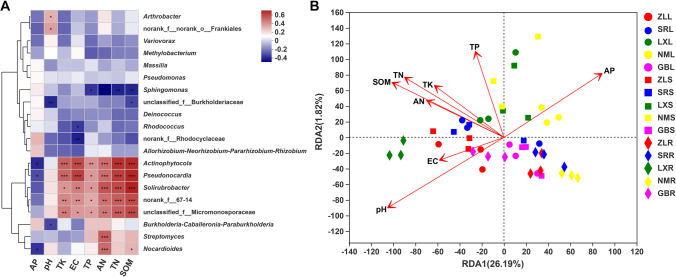
The effects of environmental factors on bacterial communities. **A** Spearman correlation analysis between the top 20 bacterial genera and environmental factors using R (version 3.3.1). **B** RDA biplot between the bacterial communities of leaf (L), stem (S), and root (R) tissue of *M. himalaica* collected from five locations (ZL, SR, LX, NM, GB) and environmental factors. The angle between the lines and samples is an acute angle, which means a positive correlation between the two elements. If the angle is obtuse, it is a negative correlation. SOM soil organic matter, TN total nitrogen, AN available nitrogen, TP total phosphorus, AP available phosphorus, TK total potassium, EC electrical conductivity. Statistical significance was calculated by R (version 3.3.1). *P < 0.05; **P < 0.01; ***P < 0.001

To analyze the effect of environmental factors on bacterial community structure, a spearman correlation heatmap and RDA were conducted to illustrate the relationship between environmental factors and endophytic community structure. As shown in Fig. 3A, the concentrations of total potassium (TK), total nitrogen (TN), available nitrogen (AN), total phosphorus (TP), soil organic matter (SOM), and EC were significantly positively correlated with the abundance of *Actinophytocola*, *Pseudonocardia*, *Solirubrobacter*, norank_f_67-14, and unclassified_f_Micromonosporaceae (*P* < 0.05), while the abundance of *Sphingomonas* was significantly negatively correlated with the concentrations of total phosphorus (TP), total nitrogen (TN), available nitrogen (AN), and soil organic matter (SOM) (*P* < 0.05). pH was significantly positively correlated with the abundance of *Arthrobacter* and norank_f_norank_o_Frankiales, while it was significantly negatively correlated with unclassified_f_Burkholderiaceae and *Burkholderia-Caballeronia-Paraburkholderia* (*P* < 0.05). The abundance of *Streptomyces* and *Nocardioides* was significantly positively correlated with the concentrations of available nitrogen (AN) (*P* < 0.05). RDA showed that the effect of environmental factors on the bacterial community was different. Of the environmental factors, pH (*r*^2^ = 0.28, *P* = 0.001) and the concentration of available phosphorus (AP) (*r*^2^ = 0.21, *P* = 0.004), total phosphorus (TP) (*r*^2^ = 0.16, *P* = 0.026), total nitrogen (TN) (*r*^2^ = 0.17, *P* = 0.017), and soil organic matter (SOM) (*r*^2^ = 0.19, *P* = 0.01) significantly affected the bacterial community, while the other factors had no significant impact (Fig. 3B).

### Endophytic community function

**Fig. 4:**
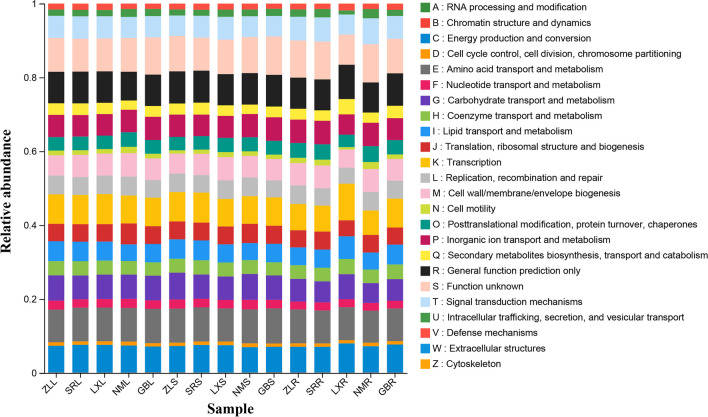
PICRUSt functional gene prediction of bacterial communities of leaf (L), stem (S), and root (R) tissue of *M. himalaica* collected from five locations (ZL, SR, LX, NM, GB). Statistical significance was calculated according to the Kruskal–Wallis *H* test. *P < 0.05; **P < 0.01; ***P < 0.001

To analyze the endophytic community functions, Clusters of Orthologous Groups of proteins (COG) analysis and KEGG functional prediction of the endophytic community were performed using PICRUST1 based on 16S rRNA sequencing data. A total of 24 COG categories were obtained, which were concentrated mainly in metabolism, such as amino acid transport and metabolism, nucleotide transport and metabolism, carbohydrate transport and metabolism, coenzyme transport and metabolism, liquid transport and metabolism, and inorganic ion transport and metabolism (Fig. 4). Likewise, the KEGG pathways with high proportions were mainly metabolism pathways, such as amino acid metabolism, carbohydrate metabolism, xenobiotic biodegradation and metabolism, energy metabolism, and lipid metabolism (Supplementary Table S1). In the leaf tissues, the relative abundances of amino acid metabolism, biosynthesis of other secondary metabolites, xenobiotic biodegradation and metabolism, and metabolism of terpenoids and polyketides were significant lower in NM than other samples (*P* < 0.05). In the stem tissues, the relative abundances of amino acid metabolism, biosynthesis of other secondary metabolites, and carbohydrate metabolism were more abundant in ZL than in LX and NM (*P* < 0.05); however, metabolism was more abundant in GB than in LX, SR, and NM (*P* < 0.05). In the root tissues, the relative abundances of biosynthesis of other secondary metabolites, carbohydrate metabolism, lipid metabolism, metabolism of terpenoids and polyketides, and xenobiotic biodegradation and metabolism in the LX were significantly higher than those in SR, ZL, and NM (*P* < 0.05). On the contrary, the relative abundances of cellular processes and signaling, environmental adaptation, glycan biosynthesis and metabolism, and infectious diseases in the NM were significantly higher than those in the LX (*P* < 0.05).

## Discussion

Endophytes are closely associated with host growth, development, stress resistance, synthesis, and pathogen biocontrol [8, 9]; they also possess the potential to synthesize medicinal compounds, which can be exploited in pharmaceuticals [37–39]. The endophytic community structure is affected by plant cultivars, plant organs, climate, altitude, and other environmental factors [40–42]. Therefore, plants growing in special climates or environments and different cultivars can be associated with novel endophytes. The endophytic community composition of *M. himalaica* was identified by high-throughput sequencing technology to illustrate the function of the core microbiota and provide a point for future research with the aim of improving production and quality of *M. himalaica*.

In the current study, we compared the richness and diversity among *M. himalaica* from five locations, and there were no certain trends in the richness of *M. himalaica* comparing samples from different tissues collected in the same place, such as, there was no significant difference in richness index between ZLL, ZLS, and ZLR, while that of SRL was significantly higher than SRS and SRR; the richness of NMR was significantly higher than NML and NMS. Likewise, similar results were observed in the bacterial diversity; the bacterial diversity parameters were higher in the SRL compared to other samples, followed by GBR, LXL, and ZLR; however, there were no significant differences between GBR, LXL, and ZLR; the bacterial diversity parameter of LXR sample was lower. The previous studies showed that the host niche, genotype, and filed location significantly affected the bacterial community composition of soybean [43]. Specific plant metabolites may influence the microbial community composition [44]. The chemical components of the wild *M. himalaica* are significantly different from those of the cultivated plant [45]. The Qinghai-Tibet Plateau has a unique geographical environment, including strong ultraviolet rays, and reports have revealed that UV-B contributes to the rotenoid biosynthesis of *M. himalaica* [46]. In addition, the microbiotas of medicinal plants are highly affected by the growth year and development stage of the plants [17, 47, 48]. Accordingly, the differences in diversity and richness might be associated with geographic conditions, growth years, and the components of secondary metabolites.

A total of 4970 OTUs were generated among the *M. himalaica* from the five locations at the 97% similarity criterion. The OTUs were classified into 35 phyla, 84 classes, 242 orders, 485 families, and 1118 genera. Proteobacteria and Actinobacteria were the dominant phyla in all the samples, and this result is in accordance with previous reports that Proteobacteria, Actinobacteria, and Firmicutes are the most common endophytes among plant samples [18, 49, 50]. However, the relative abundance of the predominant phyla was different in the different samples. Previous studies have indicated that the host niche and filed location played key roles in shaping the bacterial community structure [21, 43, 51–53]. In this article, we obtained similar results. At the top 30 genera, a total of 15 bacterial genera were significantly different among the different location-associated tissues, such as *Variovorax*, *Sphingomonas*, unclassified_f__Burkholderiaceae, *norank_f_67-14*, *Arthrobacter*, and *Solirubrobacter*. In the different tissues, a total of 15 bacterial genera were significantly different in the different tissues, such as *Deinococcus*, *Arthrobacter, Methylobacterium*, *Streptomyces*, and *Pseudonocardia*. The results indicated that host niche and filed location might influence the bacterial community.

To some extent, the leaf and stem tissues of the same location were more similar in bacterial composition. The bacterial compositions of the GB and ZL samples were more similar, while those of the LX and SR samples were more similar. *M. himalaica* is distributed mainly in dry and hot valleys, and its habitat is mainly sandy soil. The SR and LX areas have a temperate semi-humid climate type, the GB and ZL locations have a temperate humid climate type, and the NM area has plateau temperate semi-humid climate type. Previous studies showed that climate type and soil texture significantly affected microbial phyla [54]. Accordingly, we suggest that climate type had a certain impact on the community composition. In contrast to research on root tissues, except for the NM sample, other locations were more similar in community composition, which indicated that soil type may influence the bacterial community of root. RDA showed that pH, AP, TP, TN, and SOM significantly influenced the bacterial community, and these factors may shape different bacterial communities.

The core microbiota plays an important role in maintaining essential functions. In this study, we found that *Pseudomonas*, *Bacillus*, *Sphingomonas*, *Bradyrhizobium*, *Arthrobacter*, and *Streptomyces* were the core endophytic bacteria. *Pseudomonas* and *Bacillus* can promote plant growth and inhibit disease [55]. Previous studies have shown that *Bacillus* can improve the drought resistance of plants [56], which might be related to the strong drought resistance of *M. himalaica*. *Sphingomonas* can increase indole-3-acetic acid (IAA) content in soybean, promote soybean growth and resistance to osmotic stress [57], and promote plant root disease resistance, such as by enhancing lettuce root rot resistance [58], which indicates that *M. himalaica* could potentially grow well in sandy soil. *Bradyrhizobium* fixes nitrogen sources and promotes plant growth, while *Arthrobacter* and *Streptomyces* can metabolize many substances. Studies have shown that *Arthrobacter* can enhance the drought resistance of millet [59]. *Streptomyces* has been shown to reduce the effect of drought stress on maize and increase the yield of maize [60]. Thus, the strong drought resistance of *M. himalaica* might be associated with core microorganisms.

The quality of medicinal materials is related to not only environmental factors but also endophytic bacteria. Previous studies have shown that endophytic probiotics stimulate secondary metabolic ability and increase the content of medicinal components. *Bacillus*, *Piriformospora indica*, and *Azotobacter chroococcum* can increase the biomass of *Codonopsis pilosula* and *Artemisia annua* L. and promote the accumulation of medicinal components [61, 62]. PICRUSt functional prediction analysis showed that the relative abundance of bacteria is mainly involved in metabolism in the roots of *M. himalaica* from different locations. Secondary metabolites such as flavonoids, quinones, and glycolipids are the main medicinal components of *M. himalaica* [2, 5]; these pathways may contribute to the synthesis, accumulation, and transport of medicinal components of *M. himalaica*. The analysis of the KEGG pathway illustrated that the relative abundances of biosynthesis of other secondary, lipid metabolism, and metabolism of terpenoids and polyketides in the LXR sample were significantly higher than those of SRR, ZLR, and NMR samples. These results showed that the ecosystem type, climate type, and soil type may affect the synthesis of medicinal components through influencing the microbiology community.

In summary, the bacterial community composition and diversity of *M. himalaica* were investigated for the first time. The diversity and richness were significantly different in different locations and tissues. The geographic conditions, climate type, soil type, ecosystem type, and tissues determined the endophytic bacterial composition and relative bacterial abundances. Further studies are needed to understand the relationship between the endophytic community and medicinal components and environmental factors. Additionally, the function of the core microbiota needs further study with culture-independent methods to explore the potential for improving the production and quality of *M. himalaica*.

### Supplementary Information

Below is the link to the electronic supplementary material.

Supplementary file1 (DOCX 162 KB)

Supplementary file2 (DOCX 207 KB)

Supplementary file3 (DOCX 134 KB)

Supplementary file4 (DOCX 138 KB)

## Data Availability

The datasets generated during the current study are available in the NCBI-SRA repository.
