# Supplementary material for: Endophytic bacterial community structure and diversity of the medicinal plant Mirabilis himalaica from different locations
Source: Braz J Microbiol. 2023 Nov 3;54(4):2991–3003. doi: 10.1007/s42770-023-01149-1 (PMC10689605; doi:10.1007/s42770-023-01149-1)
Supplement: Supplementary file 1 — Supplementary file1 (DOCX 162 KB) [file 42770_2023_1149_MOESM1_ESM.docx]

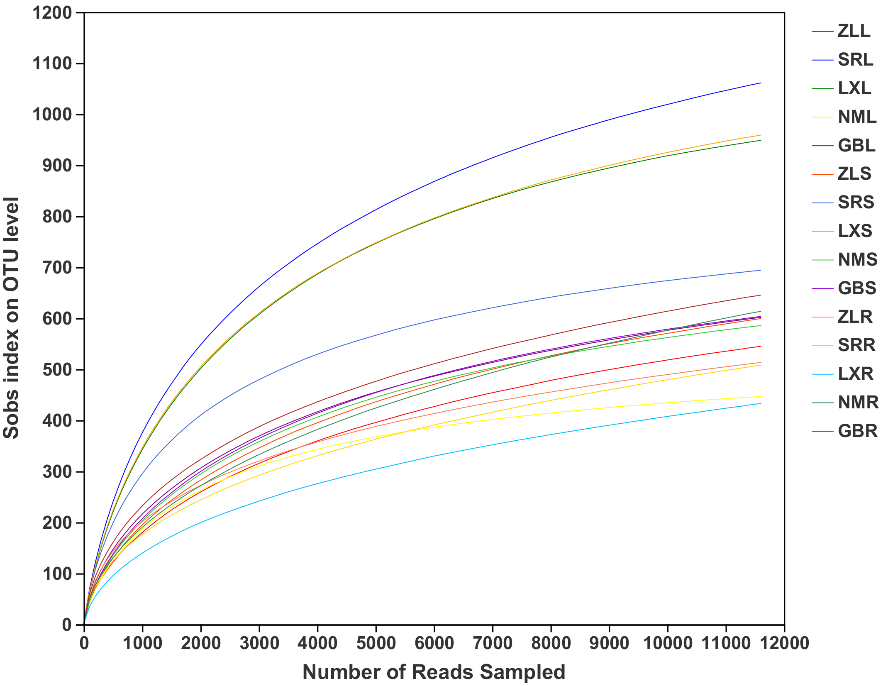


Fig. S1 Rarefaction curves of leaf (L), stem (S) and root (R) samples collected from five locations (ZL, SR, LX, NM, GB) at 97% sequence similarity
