# Supplementary material for: Endophytic bacterial community structure and diversity of the medicinal plant Mirabilis himalaica from different locations
Source: Braz J Microbiol. 2023 Nov 3;54(4):2991–3003. doi: 10.1007/s42770-023-01149-1 (PMC10689605; doi:10.1007/s42770-023-01149-1)
Supplement: Supplementary file 2 — Supplementary file2 (DOCX 207 KB) [file 42770_2023_1149_MOESM2_ESM.docx]

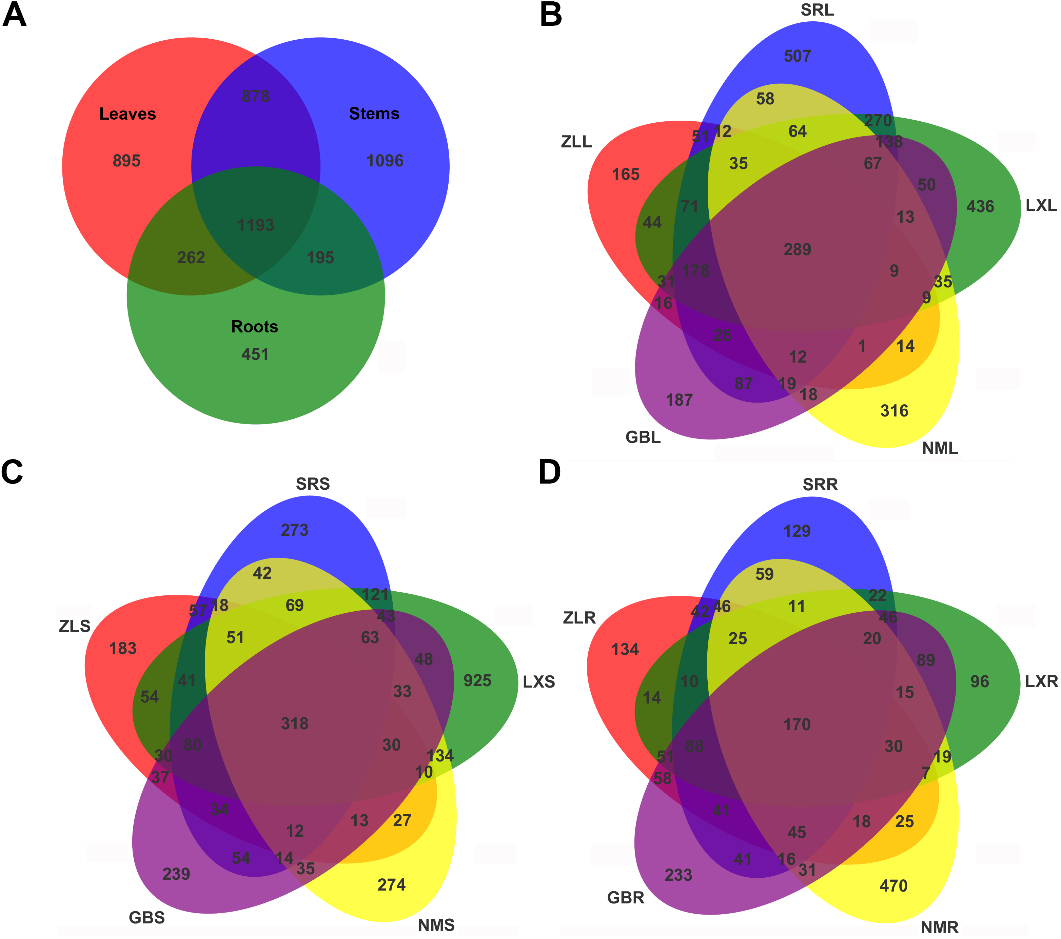


Fig.S2 Venn diagrams of the shared and unique OTUs of leaf (L) stem (S), and root (R) tissue of *M. himalaica* collected from five locations (ZL, SR, LX, NM, GB). (A) Different tissues collected from five locations. (B) Leaf tissues collected from different locations. (C) Stem tissues collected from different locations. (D) Root tissues collected from different locations. ZL: Zhangnang county; SR: Sangri county; LX: Lang county; NM: Nongmu college; GB: Gongbujiangda county.
