# Supplementary material for: Endophytic bacterial community structure and diversity of the medicinal plant Mirabilis himalaica from different locations
Source: Braz J Microbiol. 2023 Nov 3;54(4):2991–3003. doi: 10.1007/s42770-023-01149-1 (PMC10689605; doi:10.1007/s42770-023-01149-1)
Supplement: Supplementary file 3 — Supplementary file3 (DOCX 134 KB) [file 42770_2023_1149_MOESM3_ESM.docx]

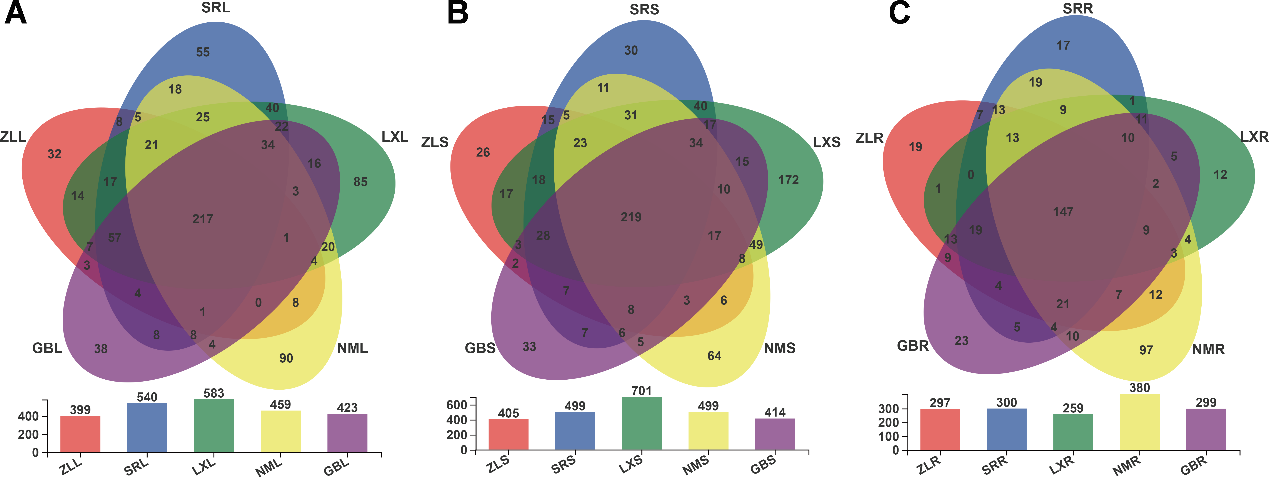


Fig. S3 Venn diagrams at the genus level of the endophytic bacteria of leaf (L) (A), stem (S) (B) and root (R) (C) tissues collected from five locations (ZL, SR, LX, NM, GB). ZL: Zhangnang county; SR: Sangri county; LX: Lang county; NM: Nongmu college; GB: Gongbujiangda county.
