# Supplementary material for: Endophytic bacterial community structure and diversity of the medicinal plant Mirabilis himalaica from different locations
Source: Braz J Microbiol. 2023 Nov 3;54(4):2991–3003. doi: 10.1007/s42770-023-01149-1 (PMC10689605; doi:10.1007/s42770-023-01149-1)
Supplement: Supplementary file 4 — Supplementary file4 (DOCX 138 KB) [file 42770_2023_1149_MOESM4_ESM.docx]

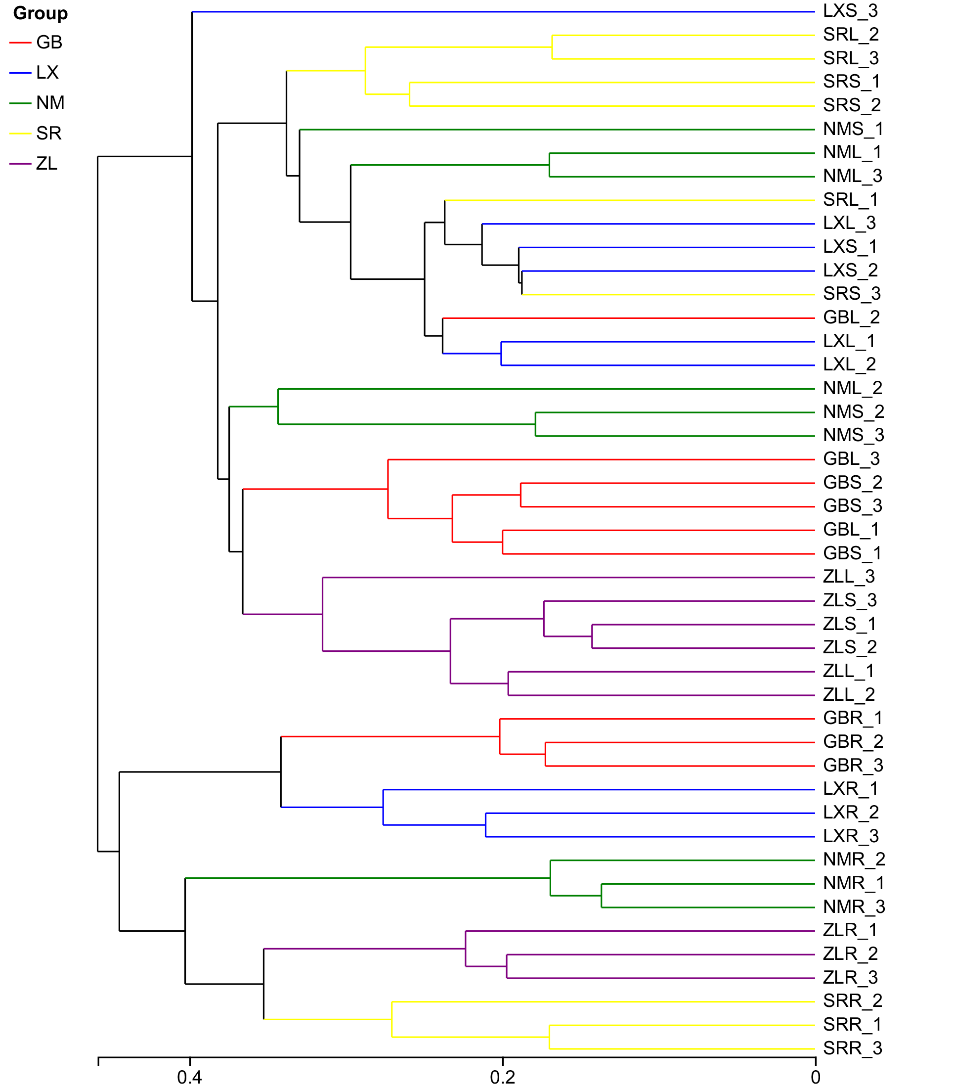


Fig. S4 UPGMA cluster analysis at the OTU level of the endophytic bacteria of leaf (L), stem (S) and root (R) tissues collected from five locations (ZL, SR, LX, NM, GB). ZL: Zhangnang county; SR: Sangri county; LX: Lang county; NM: Nongmu college; GB: Gongbujiangda county.
